# Fibrous Structures from Starch and Gluten

**DOI:** 10.3390/polym14183818

**Published:** 2022-09-13

**Authors:** Jan M. Bühler, Atze Jan van der Goot, Marieke E. Bruins

**Affiliations:** 1 Food & Biobased Research, Wageningen University & Research, Bornse Weilanden 9, 6708 WG Wageningen, The Netherlands; 2 Food Process Engineering, Agrotechnology and Food Sciences Group, Wageningen University & Research, Bornse Weilanden 9, 6708 WG Wageningen, The Netherlands

**Keywords:** starch gelatinization, thermal treatment, faba bean meal, plant-based food, tensile strength

## Abstract

Starch is added to meat analogues for binding and water holding. In this study, we investigate whether starch can have an additional role as a structuring agent. Therefore, different types of starch were combined with wheat gluten at various amounts and sheared in a High Temperature Shear Cell to determine how starch influences the structuring behavior of gluten–starch blends. The starches were chosen based on their diverse amylose contents, leading to different technological properties. Remarkable differences were found between the starches investigated. The addition of Amioca starch (containing 1% amylose) had a strong negative influence on the ability of gluten to form fibers. Maize starch (25% amylose) and Hylon VII (68% amylose) formed fibrous materials up to high starch additions. The pre-gelatinizing of maize starch further increased the ability of gluten–starch mixtures to form fibrous structures. The influence of the different types of starch on the hardness, deformability, and stiffness of the sheared samples was also assessed, revealing a spectrum of achievable properties through the addition of starch. Most remarkable was the formation of a material with anisotropy in Young’s modules. This anisotropy is also found in chicken meat, but not in protein-based fibrous materials. Furthermore, it was observed that the pre-gelatinization of starch facilitated fiber formation. A similar effect of pre-gelatinizing the starch was found when using faba bean meal with added wheat gluten, where fibrous structures could even be formed in a recipe that previously failed to produce such structures without pre-treatment.

## 1. Introduction

In a world with an ever-growing population, meat analogues are gaining more and more momentum to contribute to a more sustainable diet, and this momentum is not expected to stop [1,2,3,4,5]. The number of products on the market mimicking meat and delivering protein-rich alternatives is increasing. To make these products more attractive, they should closely resemble the fibrous structure found in real meat [6,7]. In general, a fibrous structure is regarded as a necessity for consumers to accept plant-based meat analogues as a viable alternative for real meat [8,9,10]. Fibrous structures in plant-based products can be created using the combination of heat and shear, either with an extruder or with a High Temperature Shear Cell (HTSC). Generally, these products are made from dry, plant-based, protein-rich ingredients combined with water. The exact mechanism for how these fibrous structures are created is still not fully understood, but the consensus is that a multi-phase mixture with specific properties is necessary [11,12,13,14]. The properties influencing the process have been heavily researched [10,15,16,17], along with the role of ingredients, e.g., protein–protein interactions [18,19,20] or the influence of some carbohydrates [21,22].

Currently, intensive fractionation/separation processes are used to purify protein-rich crops for plant-based meat analogues. However, these processes take away (part of) the positive effects on the sustainability of replacing animal products with plant-based products, as processing requires significant amounts of water and energy. Therefore, a transition from purified ingredients to functional fractions will be necessary in the future to keep the environmental impact of plant-based meat replacers to a minimum [23]. For starch-rich crops, this will inevitably introduce more starch to the mixture. To investigate the possibilities of using starch-rich crops, especially pulses, in meat analogues, it is important to understand the influence of starch in these protein/starch composites [24]. The exact mechanism of the effects of starch on fiber formation is not very well explored. For the HTSC, the influence of starch has not been investigated, to our knowledge. The effect of starch on protein extrudates has been studied to some extent, focusing on product properties such as hardness, chewiness, and springiness, but also fibrous degree [25,26,27,28,29]. However, these studies do not explore the mechanism of the influence of starch on fiber formation [24].

To fill this gap, dedicated studies using starches with different properties are necessary to understand their influence on fiber formation. The (functional) properties of starch are not exclusively, but significantly defined by its amylose/amylopection composition. In this study, we explore the role of starch in fiber formation in the HTSC. Special emphasis will be on the opportunities to utilize the unique gelling behavior of starch to modify product properties. We investigate the influence of three different types of starch on fiber formation, tensile strength, deformability, and the stiffness of gluten–starch blends. The starches used are chosen for their different amylose contents, leading to different technological properties: a low amylose starch (Amioca, 1%, AS), an intermediate amylose starch (maize starch, 25%, MS), and a high amylose starch (Hylon VII, 68%, HVII). Their characteristics are also heavily studied and reasonably well reported in the literature. Using starches with different amylose content to vary the properties of starch enables us to exclude factors such as the crop source of the starch, as all three starches used are variants of maize starch. Furthermore, we pre-gelatinized the intermediate amylose starch and used it in the same way as the other starches. Finally we test our findings in a recipe with gluten and faba bean meal (FM), an exemplary unrefined, starch-rich crop. FM was pre-gelatinized and processed in the HTSC in its native and pre-treated form, in combination with gluten, to transfer the findings on isolated starch to a less refined ingredient. Faba bean was chosen as it is a starch-rich crop, the isolated protein of which is suitable for the formation of a fibrous structure in the HTSC when combined with gluten [13], whereas the unrefined, starch-rich FM is not.

## 2. Materials and Methods

### 2.1. Materials

Vital wheat gluten (WG) was obtained from Roquette (Lestrem, France) and had a protein content of 74.6% w.b. (Kjeldahl, N-conversion factor = 6.25) and a dry matter content of 93.9%. Amioca starch (AS), maize starch (MS), and Hylon VII (HVII) were provided by Ingredion (Hamburg, Germany), and had an amylose content of 1%, 25%, and 68%, respectively. Faba bean meal (FM) was also provided by Ingredion (Hamburg, Germany) and had a protein content of 28.6% (Dumas, N-conversion factor = 5.7). The composition of the materials is listed in Table 1. Sodium chloride (NaCl) was acquired from Sigma-Aldrich (Zwijndrecht, The Netherlands). All components were kept at room temperature unless stated otherwise.

### 2.2. Pre-Gelatinization

Pre-gelatinized maize starch (PMS) and pre-heated FM were obtained by creating 33.3% (w.b.) MS or FM slurries in milliQ water, placing it in a sealed plastic bottle, and heating it for 60 min at 90 ${}^{{}^{\circ}}C$ in a shaking water bath. The obtained gel was kept in the sealed bottle at 4 ${}^{{}^{\circ}}C$ until use, but for at least 24 h. The dry matter content of PMS was determined to be 33.1% (w.b.) using oven drying at 105 ${}^{{}^{\circ}}C$ for 48 h.

### 2.3. High Temperature Shear Cell

Doughs containing demineralized water, NaCl, gluten, and starch (MS, PMS, AS, or HVII) or FM were prepared. The doughs were prepared following a protocol previously reported by Grabowska et al. [30], with some modifications to the temperature used based on findings of Cornet et al. [13], and to the dry matter content based on preliminary experiments performed on gluten doughs in our lab. The overall dry matter content for the samples containing starch was kept constant at 40% (w.b.), while the gluten and added starch content were varied to obtain doughs with 0–70% starch (d.b.). For the samples containing FM, the overall dry matter content was also 40%, with a WG:FM ratio of 1:1. The moisture content of the powders was taken into account when calculating the dry matter content. All doughs contained 1% (w.b.) NaCl, which was dissolved in demineralized water. The appropriate amounts of FM or starch (MS, PMS, AS, or HVII) were added and stirred by hand with a spatula until homogeneous. Gluten was added, followed by further mixing. The doughs were immediately placed in the HTSC, which was pre-heated to 140 °C. The doughs were sheared for 15 min and at 30 rpm (39 s^−1^) at 140 °C. The HTSC was cooled down to 60 °C before opening and removing the sample. The samples were placed in plastic bags, sealed, and allowed to cool to room temperature before further analysis. The sample without starch was repeated on every experiment day to monitor the reproducibility of the results throughout the experiment.

### 2.4. Assessment of the Fibrous Structure

The fibrous structure of the samples was assessed visually following a bending procedure for HTSC samples previously described by Cornet et al. [13], revealing the potential orientation of the structure in the outer 4 cm of the sample.

### 2.5. Tensile Strength Analysis

The stiffness (Young’s Modulus), maximum tensile strength (true fracture stress), and maximum deformation (fracture strain) in the parallel and perpendicular directions to the shear flow were obtained through tensile tests with a texture analyzer (TA.XTplusC, Stable Micro Systems, Surrey, UK). The tests were performed closely following a protocol previously described by Schreuders et al. [17]. In short, six dog bone-shaped tensile bars were cut from each HTSC sample, three perpendicular and three parallel to the shear flow direction. The tensile bars were clamped in the texture analyzer with a distance of 15.5 mm between the clamps. A uniaxial tensile test was performed with a displacement rate of 1 $mms^{-1}$. The force-displacement curves obtained were used to determine the true Hencky’s stress (σ) and strain (ε), which are defined as

(1) $$\varepsilon \left(t\right)=ln\frac{h(t)}{h_{0}}$$

(2) $$A\left(t\right)=\frac{h_{0}}{h(t)}*A_{0}$$

(3) $$\sigma \left(t\right)=\frac{F(t)}{A(t)}$$

where $h_{0}$ is the initial length of the sample (15.5 mm), h(t) is the length of the sample at the time of fracture, and $A_{0}$ is the initial cross-sectional area of the sample, A(t) is the cross-section area of the sample at time of fracture, and F(t) is the force at time the time of fracture. The volume of the tensile bar is assumed to remain constant throughout the measurement. The fracture stress ($\sigma_{f}$) and fracture strain ($\varepsilon_{f}$) are defined as σ and ε at the time of fracture, which is defined as the first substantial decrease in the force in the force-displacement curve. The Young’s modulus is taken as slope of the initial, linear part of the σ over ε curve (∼0.05–0.15 of the fracture strain).

## 3. Results

### 3.1. Fibrous Structure

**Figure 1:**
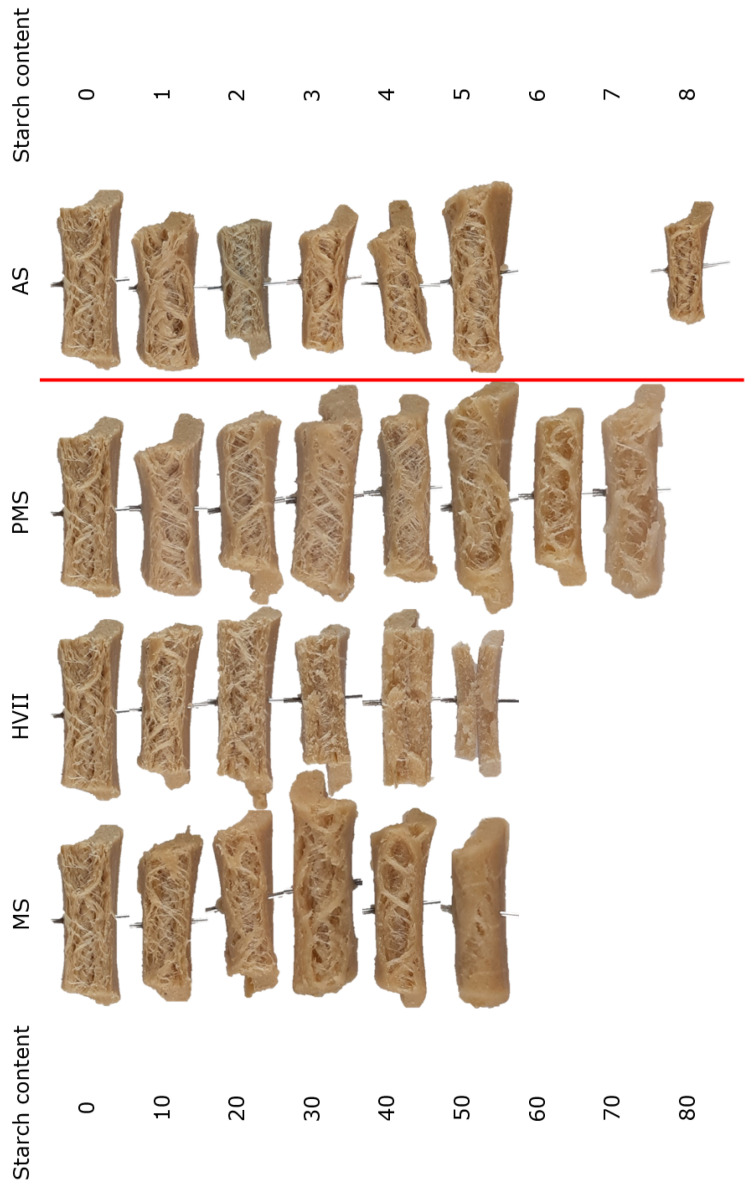
Photographs of the macro-structures after shearing MS, HVII, PMS, or AS in combination with gluten in the HTSC. Numbers in the left column indicate the amount of added starch (d.b.). The amount of added AS was 10-fold smaller, ranging over 1–8%. All samples had a dry matter content of 40% (w.b.). Each sample had a width of approximately 5 cm.

To study the effect of starches on fiber formation, we tested different starches at different concentrations in the gluten–starch blends. Gluten doughs with increasing amounts of maize starch (MS), pre-gelatinized maize starch (PMS), Amioca starch (AS), or Hylon VII (HVII) were sheared in the HTSC. The sheared samples were bent in parallel to the shear flow direction, as described by Cornet et al. [13], to visualize any fiber formation (Figure 1). The starch content was increased until no fibrous structure was visible anymore or the sample did not gel properly.

Samples without added starch exhibited a fibrous structure, meaning that the short and long fibers being oriented in the shear flow direction were visible upon bending the sample. This fibrous structure is used as a point of reference for all samples with added starch. The fibrous structure faded with increased amounts of MS, PMS, and HVII in the blend. The nature of the fibrous structure noticeably changed when ≥20% MS was added, as the number of fibers seemed to decrease and they became less distinguishable. At 40% MS, the fibrous structure was hardly noticeable anymore, while the sample containing 50% MS was too soft to break upon bending and therefore, no fibrous structure could be observed. Samples containing HVII also showed a noticeably different fibrous structure, which became less visible at 30% HVII and disappeared at higher HVII contents. Contrary to the sample containing 50% MS, the samples containing 50% HVII broke upon bending, but exhibited no fibrous structure. The addition of HVII also appeared to make the samples more stiff and brittle when bending. Samples containing any amount of PMS appeared to have smaller sized fibers, but they were still noticeable at higher added starch contents of up to 60% PMS. The maximum amount of starch that could be added without completely losing the fibrous structure ($S_{max}$) was 30%, 40%, and 60% for HVII, MS, and PMS, respectively. The addition of AS gave very different results: Samples containing >5% AS did not gel properly and could therefore not be analyzed further, with the exception of 8% AS. Remarkably, a samples containing 8% AS still exhibited a fibrous structure similar to that of samples without added starch, though they were softer and fell apart more easily.

### 3.2. Fracture Strain $\varepsilon_{f}$

**Figure 2:**
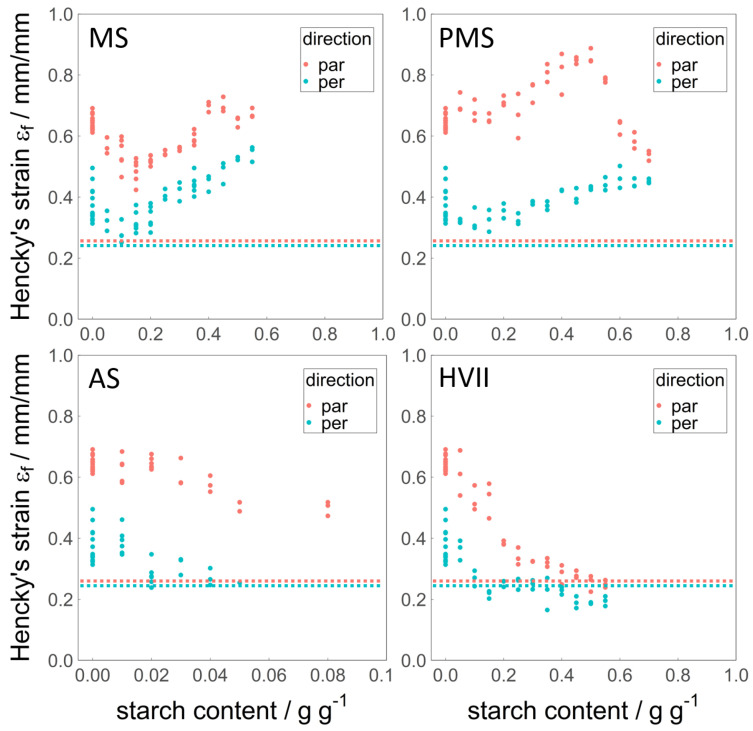
Fracture strain $\varepsilon_{f}$ in parallel (red) and perpendicular (blue) directions over the added amount of MS, PMS, AS, and HVII (d.b.). The horizontal dotted lines represent the values for cooked chicken meat, data from Schreuders et al. [17]. Note that the *x*-axis for AS has a different range than the others (0–0.1 $g\;g^{-1}$ instead of 0–1 $g\;g^{-1}$).

The fracture strain $\varepsilon_{f}$ was obtained from the tensile strength analysis and is used as a measure for the deformability of the sheared samples. It is displayed in Figure 2. The average $\varepsilon_{f}$ of the pure WG reference was 0.64 in the parallel and 0.37 in the perpendicular direction. It is therefore anisotropic, meaning that this property depends on the direction that it is measured in, or that it is not the same in every direction. This confirms the visually observed fiberous structure of the said sample. The overall values were higher than those of chicken meat, obtained by Schreuders et al. [17], which were also not anisotropic. The addition of MS caused an increase in $\varepsilon_{f}$ in both the parallel and perpendicular directions, after a small decrease at low MS addition. At $S_{max}$ (40%), the $\varepsilon_{f}$ in both directions was slightly higher than the reference. The loss of fibrous structure at MS contents >40% is not represented by the anisotropy of the deformability, since one would expect less anisotropy when there is no structural orientation. The increased deformability could be an explanation for the fact that the bending test was not able to reveal a fibrous structure at 50% MS: The material was not deformed enough to fracture in a way that the fibrous structure became visible.

The effect of the addition of PMS is peculiar: The increase in $\varepsilon_{f}$ in the perpendicular direction was less than for the addition of MS, while in the parallel direction, it was much larger and did not show the initial decrease seen for MS. This increased the anisotropy of the $\varepsilon_{f}$ up to an addition of 50% PMS. This was not expected, since the composition of this material is identical to the composition of MS. However, at $S_{max}$ (60%), the $\varepsilon_{f}$ in the parallel direction decreased sharply, arriving at values comparable to the addition of 60% MS. This shows that via pre-processing, the structuring potential of materials can be altered.

An addition of up to 5% AS resulted in a decrease in $\varepsilon_{f}$ in a similar manner to the initial decrease upon the addition of MS. Samples containing a higher amounts of AS were not suitable for tensile strength analysis, with the exception of the 8% AS sample in a parallel direction.

The addition of HVII resulted in a decrease in $\varepsilon_{f}$ in both the parallel and perpendicular directions. Unlike for the addition of MS, the decrease also persisted at higher amounts of HVII. The $\varepsilon_{f}$ at $S_{max}$ (20%) in both directions was lower than the reference, while the anisotropy was also reduced. This is in line with the observation that the samples appeared to become more brittle with the addition of HVII. HVII was the only starch that brought the deformability of the samples closer to that of cooked chicken meat.

### 3.3. Fracture Stress $\sigma_{f}$

**Figure 3:**
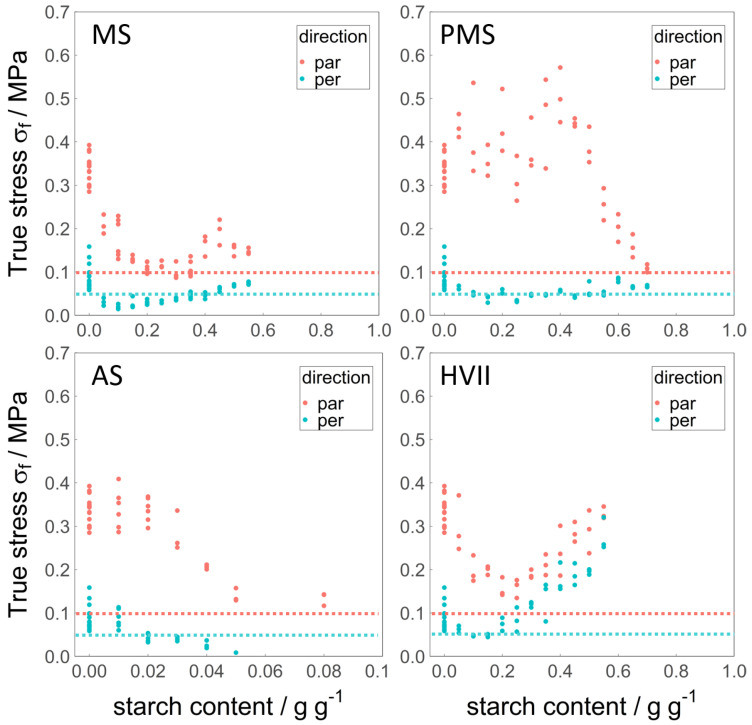
Fracture stress $\sigma_{f}$ in the parallel (red) and perpendicular (blue) directions over the added amount of MS, PMS, AS, and HVII (d.b.). The horizontal dotted lines represent the values for cooked chicken meat, data from Schreuders et al. [17]. Note that the *x*-axis for AS has a different range than the others (0–0.1 $g\;g^{-1}$ instead of 0–1 $g\;g^{-1}$).

The fracture stress $\sigma_{f}$ was obtained from the tensile strength analysis, and is used as a measure for the hardness of the sheared samples. It is displayed in Figure 3. The average $\sigma_{f}$ of the pure WG reference was 0.34 MPa in the parallel and 0.09 MPa in the perpendicular direction, and therefore, also anisotropic. This was higher overall than the fracture stress of cooked chicken meat [17], though the anisotropy is comparable. This confirms the visually observed fibrous structure of said sample.

The $\sigma_{f}$ in the perpendicular direction decreased, due to an addition of up to 10% MS, above which it increased again, returning to a similar value at an addition of 55% MS as the reference. The $\sigma_{f}$ in the parallel direction decreased rapidly, due to an addition of up to 15% MS. Above 15% MS, $\sigma_{f}$ in a parallel direction remained constant, apart from some fluctuation at higher MS addition. As a result, the anisotropy of $\sigma_{f}$ remained rather constant upon the addition of MS. There was no indication of a loss of fibrous structure at $S_{max}$ (40%). Overall, an addition of 15% MS or more resulted in a similar $\sigma_{f}$ than that of cooked chicken meat.

The effect of the addition of PMS on $\sigma_{f}$ in the perpendicular direction was the same as that of the addition of MS. In the parallel direction, the pre-gelatinization modified the effect that the starch had on $\sigma_{f}$: The values increased on average, but remained scattered up to an addition of 50% PMS, above which a sharp decrease was observed. The difference between MS and PMS in the range of 5–50% added starch is remarkable. The anisotropy of $\sigma_{f}$ in this range of added PMS was larger than for any other material or property, but was reduced at higher amounts of added PMS. Therefore, there is an indication of structure change at $S_{max}$ (60% PMS). It is possible that starch becomes the continuous phase at that concentration. The values of $\sigma_{f}$ for MS and PMS in both the parallel and perpendicular directions were remarkably similar for the corresponding $S_{max}$ (40% MS, 60% PMS).

The $\sigma_{f}$ in the parallel and perpendicular directions decreased due to the addition of small amounts of AS, comparable to the decrease due to the addition of MS. Larger amounts of added AS led to samples that were too weak to perform the tensile strength analysis on. It seems that their $\sigma_{f}$ decreased so much that they fractured under their own weight.

In contrast to the other starches, the addition of HVII led to an increase in $\sigma_{f}$ in the perpendicular direction, reaching a value similar to the initial $\sigma_{f}$ of the reference in the parallel direction. In the parallel direction, the $\sigma_{f}$ first decreased with the addition of HVII, reducing the anisotropy to a minimum. Above 25% added HVII, the $\sigma_{f}$ in both directions increased with a similar slope, keeping the anisotropy limited. This is an indication of a structure change away from fibers at $S_{max}$, as determined by visual observation.

### 3.4. Young’s Modulus

**Figure 4:**
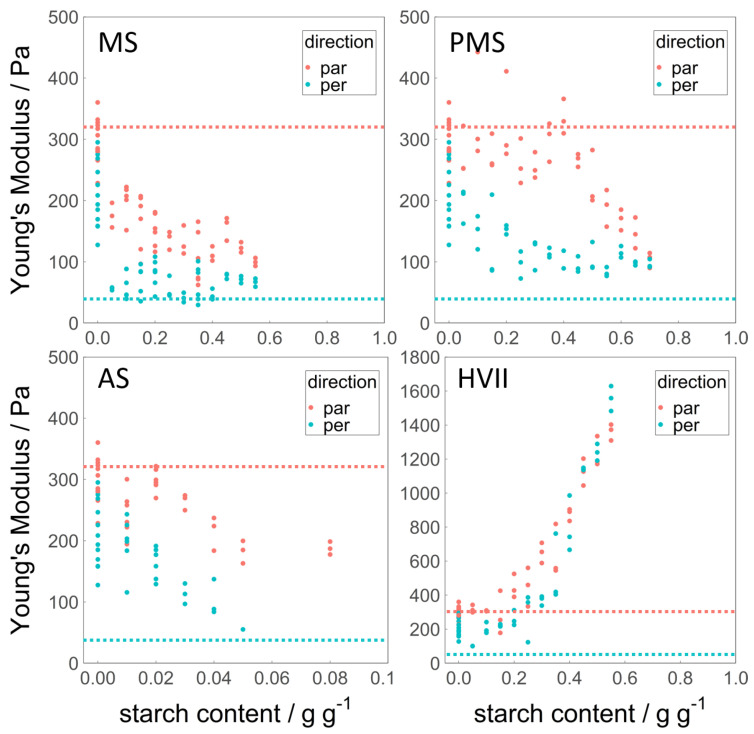
Young’s modulus in parallel (red) and perpendicular (blue) directions over the added amount of MS, PMS, AS, and HVII (d.b.). The horizontal dotted lines represent the values for cooked chicken meat, data from Schreuders et al. [17]. Note that the *x*-axis for AS (0–0.1 $g\;g^{-1}$ instead of 0–1 $g\;g^{-1}$ and the *y*-axis of HVII (0–18,000 Pa instead of 0–500 Pa) have a different range than the others.

The Young’s modulus was obtained from the tensile strength analysis and is used as a measure for the stiffness of the sheared samples. This property, unlike the fracture stress $\sigma_{f}$ or the fracture strain $\varepsilon_{f}$, gives information about the initial deformation of the sample. It is displayed in Figure 4. The average Young’s modulus of the pure WG reference was 299 Pa in the parallel and 209 Pa in the perpendicular direction, and is therefore less anisotropic than $\sigma_{f}$ or $\varepsilon_{f}$. Furthermore, the Young’s modulus of the reference was scattered, particularly in the perpendicular direction. The fibrous structure observed is inhomogeneous by nature, and especially, the initial deformation in the perpendicular direction could have been influenced by local differences in fiber thickness. The Young’s modulus in the parallel direction of the gluten reference was quite similar to that of cooked chicken meat [17], while it was much higher than cooked chicken meat in the perpendicular direction. The anisotropy of the Young’s modulus of cooked chicken meat was remarkably high.

Samples containing any amount of MS showed a lower Young’s modulus than the reference. The Young’s modulus in the parallel direction decreased with an increasing addition of MS, while it was constant and lower for the perpendicular direction. There is no indication of a loss of fibrous structure at $S_{max}$ (40%). In contrast to this, the addition of PMS led to a more gradual decrease in the Young’s modulus in the perpendicular direction, while in the parallel direction, it did not alter the Young’s modulus up to additions of 50% PMS. Therefore, the anisotropy of the Young’s modulus was largest at 40% PMS. At this point, the Young’s modulus closest resembled that of cooked chicken meat. At $S_{max}$ (60%), the Young’s modulus in the parallel and perpendicular directions were similar, and therefore did not accurately predict the loss of fibrous structure at higher PMS addition. The addition of AS caused a decrease in Young’s modulus in the parallel and perpendicular directions alike, matching the values obtained for the addition of MS in the similar range (5%).

The addition of HVII had the largest effect on the Young’s modulus. It increased in the parallel and perpendicular directions, reaching values of up to eight times higher than the reference. Due to the uniform increase, the anisotropy decreased and was lost, with values overlapping in the parallel and perpendicular directions. This confirms the observation made upon bending: the addition of HVII made the sample more stiff.

### 3.5. Faba Bean

**Figure 5:**
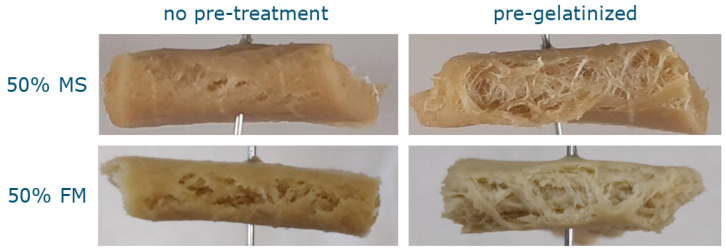
Photographs of the macrostructure after shearing gluten with untreated MS and FM, as well as pre-gelatinized MS and FM in the HTSC. All samples had a dry matter content of 40% (w.b.), with a gluten content of 50% (d.b.). Each sample has a width of approximately 5 cm.

In order to apply the findings about the influence of starch on the formation of fibrous structures to less refined ingredients, gluten was combined with untreated FM or pre-heated FM and processed in the HTSC. The sample containing 50% untreated FM did not show any fibrous structure or orientation in the shear flow direction, much like the sample containing the same amount of MS (Figure 5). The sample was soft and appeared grainy but homogeneous. The sample containing 50% pre-gelatinized FM had a fibrous structure and was soft and inhomogeneous. It seems that the pre-treatment had a similar effect on the FM as on the MS, resulting in a fibrous structure where there was none before.

## 4. Discussion

Three native starches (MS, AS, and HVII) and one pre-gelatinized starch (PMS) were added to WG at different ratios and processed in the HTSC to determine the structuring behaviors of starch–gluten blends and their rheological properties. Potential factors influencing the effect of the added starches are pH and salt content, which both depend on the purification process of the commercial starches. The pH range (pH 3.5–7 in 20% aqueous suspension) and salt content (<500 mg/100g) provided by the manufacturer are rather broad for all three starches. The pH of 5–10% aqueous solution was measured to be 4.6, 4.3, and 4.8 and the conductivity 175 μS cm^−1^, 112 μS cm^−1^, and 34 μS cm^−1^ for AS, MS, and HVII, respectively. The pH of the doughs after addition of gluten was determined for selected samples, and was consistent at pH 6.1–6.2. The pH of these doughs is dominated by the gluten protein. The conductivity does seem to differ between the starch types, but is insignificant when considering the conductivity of the NaCl added, which is roughly 14,000 μS cm^−1^ for 1% NaCl in water [31]. Based on these measurements, the influence of pH and salt content does not seem to be a determining factor.

### 4.1. Effect of Native Starch on the Sheared Samples

**Figure A1:**
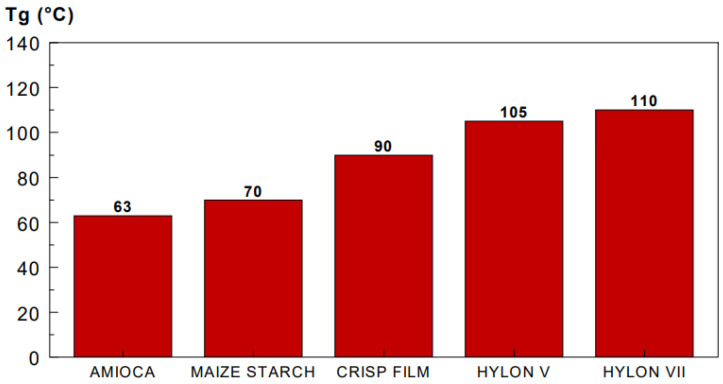
Gelling temperatures of AS (Amioca), MS (maize starch), CRISP FILM, Hylon V, and HVII (Hylon VII), measured via DSC using 20% solutions. Measurement and visualization courtesy of the manufacturer, Ingredion (Haumburg, Germany).

**Figure A2:**
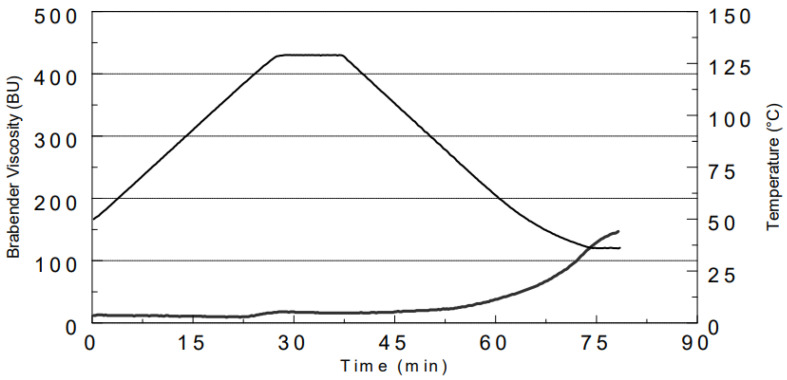
Typical Pressure Brabender Viscograph E profile of HVII using CML B264 test (5.0% starch, pH = 6.5, 130 °C max. temp.). Measurement and visualization courtesy of the manufacturer, Ingredion (Hamburg, Germany).

The three native starches that were used all contained different amylose and amylopectin contents. AS mainly contains amylopectin, and less than 1% amylose; HVII contains mostly amylose and much less amylopectin, while MS has an intermediate amylose/amylopectin content in between the two others. These differences can largely explain the rheological behaviors of the starch–gluten blends: AS and MS have similar pasting or pasting onset temperatures (Table 2) of 70–76 °C. The manufacturer measured the gelling temperature of AS to be 63 °C and that of MS to be 70 °C (see Appendix A Figure A1). The final viscosity of MS is higher than that of AS, due to the increase in amylose content. The effect of added MS and AS on the measured tensile strength, deformability, and stiffness was quite similar in the range where they could both be measured. Li et al. [32] and Ai et al. [33] investigated the gel strength of, among others, low amylose maize starch, maize starch, and high amylose maize starch after heating at 95 °C and storage. Both the low and high amylose maize starches did not form gels in their experiments. It is likely that in our study, the almost pure amylopectin in AS was unable to form a gel, leading to a loss of structure and making the samples too soft to analyze. According to the literature, HVII has a higher pasting onset temperature of 89.72–94.8 °C (Table 2), causing it to not paste completely during the RVA procedure. The final viscosity of HVII is therefore reported to be substantially lower. The manufacturer measured the gelling temperature of HVII to be 110 °C (see Appendix A Figure A1). The high temperature used in this paper allowed a complete pasting of HVII; therefore, the final viscosity is likely to be higher than what is reported in literature, due to the higher amylose content. The manufacturer measured the final viscosity of HVII to be around 150 BU (Brabender Units) after a heat treatment at 130 °C (see Appendix A Figure A2). Carvalho et al. [34] also captured this phenomenon. The recrystallization of amylose upon cooling after complete pasting caused stiffness; the deformability decreased and the hardness increased.

Fiber formation is believed to be related to the technological properties of the ingredients, including the water holding capacity [37,38] and rheological properties such as viscosity [10,30,39,40]. The starches investigated in this study differ in their amylose/amylopectin content, which leads to differences in these technological properties. Fiber formation is therefore affected by the amylose/amylopectin content of the starch: Fiber formation is easily disturbed by AS addition, as it does not form a gel after cooling. The inability of amylopectin to form a gel upon cooling [32,33] caused the samples to fall apart. Furthermore, amylopectin degrades under high temperature and shear [41], potentially drastically lowering its viscosity. This could have had a lubricating effect, preventing proper shearing of the remaining gluten part. The large effect of low addition levels of AS can be attributed to the fact that AS takes up relatively large amounts of water (Table 2), adding to its volume. While non-gluten ingredients do not actively contribute to the formation of fibrous structures in gluten containing blends and therefore act as fillers [13], they can prevent the formation of fibrous structures if they are not able to form a strong enough gel. Amylose disturbed fiber formation less, although too much amylose (in the case of HVII) apparently also prevented gluten from forming a fibrous structure, as the high amylose gel became too stiff. Furthermore, the low water retention capacity of HVII (Table 2) could have diluted the gluten phase in such a way that it prevented fiber formation. As mentioned earlier, previous studies on the fiber formation in the HTSC assumed that the rheological properties of the ingredients have an influence on fiber. Dekkers et al. [42] claimed that the rheological properties of the “phases” should be similar, while Schreuders et al. [40] hypothesized that a bi-continuous network is a requirement for fiber formation, the existence of which is governed by the rheological properties of the phases. Cornet et al. [13] concluded that a continuous gluten network was essential in order to create a fibrous structure. There, a gluten content of at least 50% was necessary to create a fibrous structure in combination with leguminous protein isolates. This is in line with our results for native starches. Potentially, the stiff and therefore less deformable nature of the amylose-rich HVII after cooling also influenced the breaking behavior, breaking the gluten fibers while bending the sample. An optimum of the softness of amylopectin and the stiffness of amylose seems to be necessary to ensure the proper embedding of the gluten fibers during shearing and bending. A high amount of amylopectin disrupted fiber formation during shearing (lubrication effect) and after cooling (low gel strength), while a high amount of amylose disrupted fiber formation after cooling (breaking behavior).

### 4.2. Effect of Pre-Gelatinization

Pre-gelatinizing MS prior to mixing with gluten and processing in the HTSC mainly modified the stiffness, strength, and deformability in the parallel direction of the shear flow, while the values in the perpendicular direction remained close to those of MS. This is likely not an additive effect, but an indirect effect of PMS on the WG and the fibrous structure. The properties measured in the parallel direction can be regarded as the properties of the gluten fibers. Pre-gelatinized starch takes up more water at room temperature than native starch. WG therefore had limited water available when mixed with the PMS slurry in the sample preparation. As gluten already forms a network when mixed with water at room temperature, the limited water content of the WG resulted in a more dense WG network. This could have led to stronger, stiffer, and more deformable WG fibers, even at high amounts of PMS addition. In the perpendicular direction, the gluten dominates at low starch contents and determines the properties. As the starch content increases, the adhesion of gluten/starch (which depends on the type of starch) starts to play a role, decreasing the strength and deformability. At higher starch contents, the starch dominates the properties; in the case of HVII, this led to a low deformability, though an increased strength. This is why the effect of pre-gelatinization, and so the difference between MS and PMS, mainly manifested in the parallel direction. The small differences in perpendicular direction could stem from the different conditions during gelatinization of the starch: while the starch in the PMS was allowed to gel freely with only water being present, the starch in MS was competing with the gluten for water and was sterically hindered by the gluten network during swelling and gelatinization. The anisotropy of the Young’s modulus largely increased with pre-gelatinization. This made the samples containing PMS best match the Young’s modulus of chicken strips. Such a resemblance of a real meat texture has not been reported before. The use of pre-gelatinized starch could be a unique option to advance the development of meat analogues that actually resemble real meat.

### 4.3. Application in Faba Bean Meal

The sample containing 50% FM did not form a fibrous structure, likely due to a limited water uptake of FM at room temperature that diluted the gluten phase. FM had a starch content of 38.5%, resulting in an added starch content of 19.25% in the sheared samples. This falls in the range of the starch contents tested that could form fibrous products. The starch of FM (40% amylose) has a higher amylose content than MS (27%), but lower than HVII (68%), so on one hand, one would expect it to form a fibrous structure up to an addition of between 30% and 40%. On the other hand, the gluten content in the sheared FM samples was 50%, a recipe that also failed to produce fibrous structures with MS and HVII. FM also contains native protein, which is generally regarded to be less suitable for fiber formation in the HTSC [38,43]. Furthermore, Li et al. [32] found that faba bean starch has a much higher gel strength than maize starch, even though their amylose content and branch-chain-length distribution, as well as the average degree of polymerization, were comparable. This suggests that the differences between starches are not fully captured by their amylose/amylopectin composition. Pre-gelatinizing the MS modified the water uptake of the starch at room temperature, resulting in a denser gluten network and a fibrous structure at the same amount of added starch. This concept turned out to be also applicable to less pure ingredients than commercial starch, since the same effect of pre-gelatinization was observed for FM. This shows that less refined materials, thought to be less suitable for the formation of fibrous structures, can be modified “in-situ”, and so without the separation of the components. Due to the difference of the temperature at which thermal transitions occur in the starch (73.4 °C) and protein (>120 °C) of faba bean at the moisture content used, it is possible to gelatinize the starch in FM without denaturing the protein [44]. This approach was previously suggested by Bühler et al. [24]. This proof of concept is likely to be expandable to other starch-rich crops, such as peas or lentils.

## 5. Conclusions

The effect of the addition of starch on the structural formation of gluten in the HTSC was investigated. It is possible to add substantial amounts of native starch (up to 40%) to gluten and still obtain a fibrous structure. With the addition of starch, the textural properties of the samples change depending on the type and amount of starch, which can result in textural properties not previously seen for protein blends. The differences in the amylose/amylopectin contents of the starches lead to different technological properties such as viscosity and water uptake, thereby affecting the fiber formation and textural properties. Pre-gelatinization of the starch enhances the ability of the starch/gluten blends to form fibrous products, allowing for the addition of up to 60% of starch. It also leads to an increased anisotropy of the Young’s modulus, resembling chicken meat. This effect, and the higher maximum addable amount of PMS, are attributed to the higher water uptake of PMS at room temperature, which increases the density of the gluten network and therefore strengthens it. This concept was applied to FM, a unrefined material containing a considerable amount of starch. It was shown that not only can starch can be used to move the properties of plant-based meat analogues closer to those of real meat, but unrefined, starch-rich crops can be modified “in-situ” to become suitable for the production of fibrous meat analogues. 

## Figures and Tables

**Table 1:** Composition of the raw materials.

	Dry Matter/%	Protein Content /%	Starch Content /%
FM	-	28.6 ^1^	38.5 ^2^
WG	93.9 ^3^	74.6 ^4^	12.6 ^5^
MS	91.9 ^3^	-	>97 ^6^
AS	93.5 ^3^	-	>97 ^6^
HVII	92.0 ^3^	-	>97 ^6^

^1^ Dumas (Nx5.7). ^2^ Megazyme Starch Kit. ^3^ Determined via oven drying. ^4^ Kjeldhal (Nx6.25). ^5^ Chromatography sugar analysis. ^6^ Product data sheet, Ingredion.

**Table 2:** Properties of Amioca, maize starch, and Hylon VII from the literature. It is important to remark that the properties in this table are measured at lower temperatures than those used in our experiments, and HVII does not yet paste fully, causing the reported low viscosity measurements for HVII.

	Amioca	Maize	Hylon VII
amylose/%	<1	27	68
pasting temp. (RVA)/°C	72.68 [35]	76.12 [35]	-
pasting temp. (DSC)/°C	71.98 [36]	73.81 [36]	89.72 [36]
	71.2 [32]	70.1 [32]	-
onset temp. (RVA)/°C	70.2 [34]	72.3 [34]	94.8 [34]
final visc. (RVA)/mPas	2349 [34] ^7^	2546 [34] ^7^	46 [34] ^7^
	2191 [35]	3153 [35]	<1 [35]
water retention capacity at 90 °C/g/100 g	20.40 [36]	11.19 [36]	4.48 [36]

^7^ calculated from their data.

## Data Availability

The data presented in this study are available on request from the corresponding author.

## Abbreviations

The following abbreviations are used in this manuscript:

DSC	Differential Scanning Calorimetry
HTSC	High Temperature Shear Cell
AS	Amioca starch
MS	Maize starch
HVII	Hylon VII
PMS	Pre-gelatinized maize starch
WG	Wheat gluten
FM	Faba bean meal
